# Effect of Implant vs. Tissue Reconstruction on Cancer Specific Survival Varies by Axillary Lymph Node Status in Breast Cancer Patients

**DOI:** 10.1371/journal.pone.0118161

**Published:** 2015-02-18

**Authors:** Qian Ouyang, Liling Zhu, Kai Chen, Fengxi Su

**Affiliations:** Breast Tumor Center, Sun Yat-sen Memorial Hospital, Sun Yat-sen University, Guangzhou, Guangdong, P.R.China; Queen Mary Hospital, HONG KONG

## Abstract

**Background:**

To compare the breast cancer-specific survival (BCSS) between patients who underwent tissue or implant reconstruction after mastectomy.

**Method:**

We used the database from Surveillance, Epidemiology, and End Results (SEER) registries and compared the BCSS between patients who underwent tissue and implant reconstruction after mastectomy. Cox-regression models were fitted, adjusting for known clinicopathological features. The interaction between the reconstruction types (tissue/implant) and nodal status (N-stage) was investigated.

**Results:**

A total of 6,426 patients with a median age of 50 years were included. With a median follow up of 100 months, the 10-year cumulative BCSS and non-BCSS were 85.1% and 95.4%, respectively. Patients who underwent tissue reconstruction had tumors with a higher T-stage, N-stage, and tumor grade and tended to be ER/PR-negative compared to those who received implant reconstruction. In univariate analysis, implant-reconstruction was associated with a 2.4% increase (P = 0.003) in the BCSS compared with tissue-reconstruction. After adjusting for significant risk factors of the BCSS (suggested by univariate analysis) and stratifying based on the N-stage, there was only an association between the reconstruction type and the BCSS for the N2-3 patients (10-year BCSS of implant vs. tissue-reconstruction: 68.7% and 59.0%, P = 0.004). The 10-year BCSS rates of implant vs. tissue-reconstruction were 91.7% and 91.8% in N0 patients (P>0.05) and 84.5% and 84.4% in N1 patients (P>0.05), respectively.

**Conclusions:**

The implant (vs. tissue) reconstruction after mastectomy was associated with an improved BCSS in N2-3 breast cancer patients but not in N0-1 patients. A well-designed, prospective study is needed to further confirm these findings.

## Introduction

The aim of breast reconstruction after mastectomy is to rebuild the shape of the removed breast and maintain quality of life without affecting the oncological safety of breast cancer treatment. Studies have shown that breast reconstruction (Implant/Tissue) after mastectomy does not impair the post-operative surveillance of tumor recurrence[1,2] and does not delay any adjuvant therapies[3], compared with mastectomy alone. The evolution from prosthetic implants to autologous tissue constructs has provided more options for breast reconstruction and increased the popularity of such approaches. The advantages of implant reconstruction include a relatively short procedure time, no procedures on the donor site and fewer complications. The tissue reconstruction approach, however, has the advantages of creating a softer, more ptotic and natural-appearing breast mound[4], but it is associated with more complications. These two approaches (tissue/implant), theoretically, were supposed to have similar long-term survival. However, few population-based studies address this issue. Bezuhly et al[5] reported, using the SEER database, that immediate breast reconstruction(tissue/implant) after mastectomy (vs. Mastectomy alone) was associated with improved BCSS. They did not provide a detailed description of the effectiveness of these two approaches in their study. In this Surveillance, Epidemiology, and End Results (SEER)-based population study, we aim to study the breast cancer specific survival (BCSS) between patients who received tissue or implant reconstruction after mastectomy. We are also going to investigate whether the difference in the BCSS between the reconstruction types, if present, varies across different subgroups of patients.

## Methods

### Data collection

We searched the SEER registry data from 18 registries (Nov 2013 submission) and identified female patients with non-metastatic breast cancer between 1998–2005. The tumor grade, adjusted AJCC 6^th^ stage, adjusted AJCC 6^th^ T-stage and N-stage, surgery of the primary site, radiation, race, marital status at diagnosis, laterality (left or right breast), estrogen receptor (ER), progesterone receptor (PR), CHSDA (Contract Health Service Delivery Areas) region, survival month, county attributes (median family income, percentage of people with more than a 9^th^ grade education level, percentage of families under poverty lines and county type (metropolitan/non-metropolitan)), SEER cause-specific death classification and SEER other cause of death classification were extracted. The breast surgery code as reviewed was based on the SEER Program Coding and Staging Manual 2012. Patients who received unilateral breast tissue (Code 44,54) or implant (Code 45,55) reconstruction were included. Patients with a borderline ER and PR status were considered as ER and PR positive, respectively. County attributes (median family income, percentage of people with more than a 9^th^ grade education level, and percentage of families under the poverty line) were classified into four subgroups by the quartiles number/percentage of each index. This study used a national dataset of de-identified patient information and did not meet the Sun Yat-sen Memorial Hospital’s criteria for institutional review board (IRB) approval. Hence this study waived the need for IRB approval.

### Exclusion criteria

1. Important prognostic information, such as the grade, AJCC stage, T-stage, N-stage, surgery, radiation unknown or not specified, and ER or PR unknown;
2. Patients who received combined tissue and implant reconstructions (e.g., Code 46,56).
3. Patients who received mastectomy on the contralateral, uninvolved breast.
4. Patients who received radical mastectomy or extended radical mastectomy.
5. Patients who were registered in the Alaska Area of the CHSDA region.

### Statistical analysis

We conducted a descriptive analysis of the population characteristics. The Chi-square test was used to compare the differences of the demographical and clinicopathological features between patients who received implant or tissue reconstruction. Kaplan-Meier survival analysis was used to calculate the cumulative BCSS (death due to breast cancer) and cumulative non-BCSS (death event due to causes other than breast cancer).

In univariate analysis, we used the log-rank test, together with an unadjusted cox-regression model to screen for potential risk factors for BCSS. Significant risk factors from univariate analysis (age, race, CHSDA region, marital status, T-stage, N-stage, histology, ER, PR, grade, simple/modified radical mastectomy, radiotherapy and tissue/implant reconstruction) were incorporated into the adjusted cox-regression model as a multivariate analysis. Family income was also included in the multivariable model. Interactions between the reconstruction type (tissue/implant) and other factors (T-stage, N-stage, ER/PR status, etc) were investigated. Subgroup analysis was performed after the identification of significant interactions. In adjusted Cox-regression analysis, propensity score matching was conducted to eliminate the potential influence of “confounding by indication”. We used a logistic-regression model to generate the predicted probability (propensity score) of receiving tissue/implant reconstruction and kept it as a covariate in the adjusted Cox-regression analysis. All P-values are two-sided, and P values <0.05 were considered statistically significant. The statistical analysis was performed using Stata/SE, version 12.0 (StataCorp LP, College Station, TX).

## Results

### Population characteristics

This study included 6,426 patients with a median follow up of 100 months. The cumulative BCSS and non-BCSS were 92.0% and 98.4% at 5 years and 85.1% and 95.4% at 10 years, respectively. The median age of this population was 50. As shown in Table 1, patients who received tissue reconstruction were more likely to be African American, located in the East part (CHSDA region) of the United States and to have received modified radical mastectomy compared with patients who received implant reconstruction. Additionally, there were more patients with a higher T-stage, N-stage and tumor grade as well as ER-negative and PR-negative diseases in the tissue reconstruction cohort than in the implant reconstruction cohort. Therefore, the tumor burden was imbalanced at baseline before the two cohorts.

**Table 1:** Characteristics of patients included in this study.

Item	Tissue		Implant		P
	n	%	n	%	
**Age Groups**					
<50yrs	2,005	50.44	1,182	48.23	NS
≥50yrs	1,970	49.56	1,269	51.77	
**County Type**					
Metropolitan	3,708	93.28	2,295	93.64	NS
Non-Metropolitan	253	6.36	147	6.00	
Unk	14	0.35	9	0.37	
**Education Level, quartile**					
1st (Lowest)	1,006	25.31	670	27.34	<0.01
2nd	1,204	30.29	557	22.73	
3rd	775	19.50	628	25.62	
4th (Highest)	990	24.91	596	24.32	
**Family Income, quartile.**					
1st (Lowest)	1,200	30.19	652	26.60	<0.01
2nd	793	19.95	619	25.26	
3rd	1,002	25.21	606	24.72	
4th (Highest)	980	24.65	574	23.42	
**Race**					
White	3,335	83.90	2,153	87.84	<0.01
African American	441	11.09	147	6.00	
Others	199	5.01	151	6.16	
**CHSDA Region**					
East	1,906	47.95	752	30.68	<0.01
Northern Plains	555	13.96	406	16.56	
Pacific Coast	1,201	30.21	1,124	45.86	
Southwest	313	7.87	169	6.90	
**Marital Status**					
Married	2,748	69.13	1,682	68.63	NS
Divorced/Separated/Single/Widowed/Unknown	1,227	30.87	769	31.37	
**Laterality**					
Left	2,014	50.67	1,255	51.20	NS
Right	1,961	49.33	1,196	48.80	
**Primary Site**					
Nipple/Central Portion	271	6.82	180	7.34	NS
UIQ	363	9.13	240	9.79	
LIQ	184	4.63	133	5.43	
UOQ	1,302	32.75	754	30.76	
LOQ	263	6.62	142	5.79	
Overlapping/Unknown	1,592	40.05	1,002	40.88	
**Histology**					
Infiltrating Duct Carcinoma, Nos	2,876	72.35	1,728	70.50	NS
Lobular Carcinoma, Nos	248	6.24	189	7.71	
Infiltrating Duct And Lobular Carcinoma	462	11.62	293	11.95	
Others	389	9.79	241	9.83	
**T-Stage**					
Tmic-T1a	369	9.28	209	8.53	<0.01
T1b	487	12.25	356	14.52	
T1c	1,267	31.87	842	34.35	
T2	1,495	37.61	856	34.92	
T3-T4	357	8.98	188	7.67	
**N-Stage**					
N0	2,080	52.33	1,421	57.98	<0.01
N1	1,272	32.00	708	28.89	
N2-N3	623	15.67	322	13.14	
**Ajcc-Stage**					
I	1,405	35.35	1,001	40.84	<0.01
IIa	1,156	29.08	711	29.01	
IIb	664	16.70	349	14.24	
IIIa-C	750	18.87	390	15.91	
**ER**					
Negative	984	24.75	536	21.87	<0.01
Positive	2,991	75.25	1,915	78.13	
**PR**					
Negative	1,384	34.82	759	30.97	<0.01
Positive	2,591	65.18	1,692	69.03	
**Grade**					
I	528	13.28	412	16.81	<0.01
II	1,656	41.66	1,071	43.70	
III	1,710	43.02	923	37.66	
IV	81	2.04	45	1.84	
**Mastectomy Type**					
Simple Mastectomy	1,100	27.67	810	33.05	<0.01
Modified Radical Mastectomy	2,875	72.33	1,641	66.95	
**Radiotherapy**					
No	3,055	76.86	1,930	78.74	NS
Yes	920	23.14	521	21.26	

CHSDA,Contract Health Services Delivery Areas;UIQ, Upper-inner quadrant; LIQ, Lower-inner quadrant; UOQ, Upper-outer quadrant; LOQ, Lower-outer quadrant;ER, Estrogen receptor; PR, Progesterone receptor;Chi-square test was used.

### Risk factor for BCSS: Univariate analysis

In univariate analysis (S1 Table), we observed that African American (vs. White), age<50 yrs, Northern plains (vs. East) area, divorced/separated/single/widowed (vs. married), infiltrating ductal carcinoma (vs. lobular carcinoma), higher T-stage, higher N-stage, negative ER, negative PR, higher tumor grade, modified radical mastectomy (vs. simple mastectomy), radiotherapy, and tissue reconstruction (vs. implant) were all associated with decreased BCSS. These factors were all included in the multivariate analysis. The cumulative BCSS rates in the tissue and implant cohort were 91.4% and 93.0% at 5 years and 84.3% and 86.7% at 10 years (log-rank test, P = 0.003), respectively. The cumulative non-BCSS rates in the tissue- and implant- cohorts were 98.7% and 98.0% at 5 years and 96.1% and 94.2% at 10 years (log-rank test, P = 0.002), respectively.

### Risk factor for BCSS: Multivariate analysis

**Fig 1:**
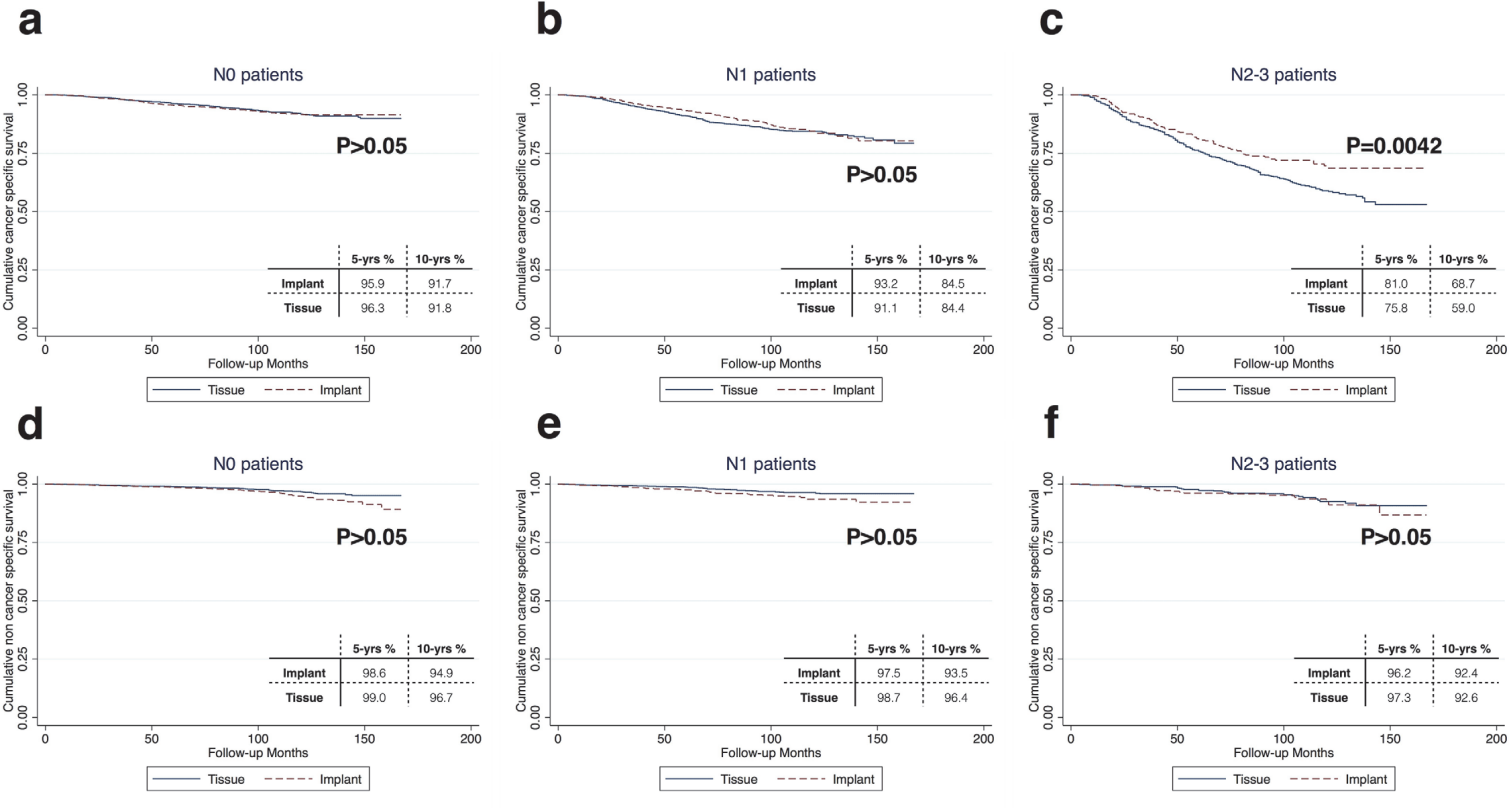
a-c, Cumulative BCSS in patients who underwent tissue and implant reconstruction; d-f, Cumulative non-BCSS in patients who underwent tissue and implant reconstruction.

In the Cox-regression model, we noticed a significant interaction between the N-stage and reconstruction type (tissue/implant) (P = 0.02, 95%CI 0.68–0.97, S2 Table). Therefore, subgroup analysis based on the N-stage was performed. As shown in Table 2, implant (vs. tissue reconstruction) was associated with a lower risk of breast cancer death in N2-3 patients (Fig. 1), after it was controlled for some other clinicopathological factors (HR = 0.76, P = 0.03, 95%CI 0.59–0.97). The reconstruction type (implant/tissue) was not associated with BCSS in patients with N0-1 diseases (Table 2 and Fig. 1). The propensity score was incorporated as a covariate in the multivariate analysis and the results remained the same (S3 Table).

**Table 2:** Adjusted Cox-regression in patients with different tumor burden in the axilla.

Variable	N0		N1		N2-N3	
	HR (95%CI)	P	HR (95%CI)	P	HR (95%CI)	P
**Age groups**						
<50yrs	1.00		1.00		1.00	
≥50yrs	0.95(0.73–1.22)	0.67	0.93(0.73–1.18)	0.53	0.95(0.75–1.19)	0.65
**Family Income, quartile.**						
1st (Lowest)	1.00					
2nd	1.03(0.72–1.48)	0.88	0.89(0.62–1.27)	0.51	1.47(1.08–1.99)	0.01
3rd	1.08(0.77–1.51)	0.67	1.08(0.78–1.49)	0.64	0.87(0.64–1.20)	0.41
4th (Highest)	1.16(0.81–1.66)	0.43	1.12(0.81–1.57)	0.49	0.93(0.67–1.29)	0.66
**Race**						
White	1.00		1.00		1.00	
African American	1.181(0.78–1.79)	0.44	1.54(1.09–2.16)	0.01	1.16(0.83–1.62)	0.39
Others	0.56(0.27–1.14)	0.11	1.17(0.70–1.95)	0.55	0.78(0.42–1.45)	0.42
**CHSDA Region**						
East	1.00		1.00		1.00	
Northern Plains	1.53(1.07–2.19)	0.02	1.00(0.71–1.42)	0.99	1.55(1.11–2.16)	0.01
Pacific Coast	1.23(0.89–1.69)	0.21	1.03(0.77–1.38)	0.85	0.78(0.59–1.04)	0.09
Southwest	1.10(0.65–1.87)	0.72	1.73(1.07–2.78)	0.03	1.13(0.75–1.71)	0.56
**Marital status**						
Married	1.00		1.00		1.00	
Divorced/Separated/Single/Widowed/Unknown	1.19(0.91–1.55)	0.20	1.29(1.00–1.66)	0.05	1.31(1.04–1.65)	0.02
**T-stage**						
T1	1.00		1.00		1.00	
T2	1.60(1.22–2.10)	<0.01	1.68(1.28–2.20)	<0.01	1.99(1.42–2.79)	<0.01
T3	2.92(1.76–4.85)	<0.01	3.00(2.05–4.39)	<0.01	2.69(1.87–3.87)	<0.01
**Histology**						
Infiltrating duct carcinoma	1.00		1.00		1.00	
Lobular carcinoma	0.56(0.27–1.15)	0.11	0.83(0.45–1.52)	0.55	0.97(0.62–1.52)	0.89
Infiltrating duct and lobular carcinoma	0.78(0.45–1.34)	0.36	0.99(0.67–1.45)	0.95	1.08(0.74–1.57)	0.70
Others	0.92(0.61–1.39)	0.70	0.97(0.62–1.51)	0.88	1.15(0.80–1.65)	0.45
**ER**						
Negative	1.00		1.00		1.00	
Positive	0.60(0.41–0.86)	0.01	0.96(0.69–1.34)	0.83	0.54(0.38–0.76)	<0.01
**PR**						
Negative	1.00		1.00		1.00	
Positive	0.72(0.50–1.02)	0.07	0.53(0.39–0.73)	<0.01	0.82(0.60–1.12)	0.21
**Grade**						
I	1.00		1.00		1.00	
II	2.19(1.23–3.88)	0.01	5.8(2.14–15.92)	<0.01	1.71(0.89–3.30)	0.11
III	3.29(1.84–5.88)	<0.01	7.40(2.70–20.25)	<0.01	1.90(0.99–3.67)	0.06
IV	2.46(0.93–6.53)	0.07	12.48(3.72–41.90)	<0.01	2.35(0.97–5.66)	0.06
**Mastectomy Type**						
Simple Mastectomy	1.00		1.00		1.00	
Modified Radical Mastectomy	1.21(0.90–1.61)	0.20	1.53(1.09–2.16)	0.01	1.03(0.69–1.54)	0.88
**Radiotherapy**						
No	1.00		1.00		1.00	
Yes	1.30(0.87–1.95)	0.20	1.03(0.79–1.34)	0.84	0.77(0.61–0.97)	0.02
**Reconstruction Type**						
**Tissue**	**1.00**		**1.00**		**1.00**	
**Implant**	**1.14(0.88–1.47)**	**0.33**	**1.00(0.78–1.29)**	**0.97**	**0.76(0.59–0.97)**	**0.03**

HR, hazard ratio; CI, confidence interval; ER, Estrogen receptor; PR, Progesterone receptor.

### Tissue and implant reconstructions in N2-3 patients

The clinicopathological features at baseline between the two approaches in N2-3 patients were similar (S4 Table). The cumulative BCSS rates in the tissue- and implant- cohorts were 75.8% and 81.0% at 5 years and 59.0% and 68.7% at 10 years (log-rank test, P = 0.004), respectively. The non-BCSS survival was similar between the two cohorts (Fig. 1).

## Discussion

### Reconstruction methods (tissue/implant) and BCSS in N2-3 patients

In this study, we noticed improvement in the 10-year BCSS in the implant reconstruction cohort (68.7%) compared with the tissue reconstruction cohort (59.0%) in N2-3 non-metastatic breast cancer patients after mastectomy. We are very cautious in interpreting these results and pay special attention to the influence of “confounding by indication” in this retrospective study:

1. We did not observe any differences in the non-BCSS between these two approaches in N2-3 patients (Fig. 1), suggesting that the choices of the reconstruction methods may not be associated with the patient comorbidities at baseline.
2. We studied the baseline characteristics of the N2-3 patients and did not observe any significant differences (e.g., T-stage, ER status and tumor grade) between the two cohorts of patients (S4 Table). This is strong evidence that this subgroup of patients may not have “confounding by indication”;
3. After adjustment for the family income, T-stage, ER status, PR status, tumor grade, surgery type, race and age, we still observed that implant-reconstruction was associated with an improved BCSS (Table 2, Fig. 1) in this subgroup of patients. Jiang et al[6] also analyzed the SEER database and showed that after adjusting for the family income in the multivariate analysis, the BCSS differences were limited between patients who underwent tissue and implant reconstructions. However, the authors did not report a significant interaction between the N-stage and reconstruction methods. In our study, the reconstruction method was significantly associated with BCSS in N2-3 patients, even after adjusting for the family income.
4. The association was still significant even after propensity score matching (S3 Table).

### Study limitations

Another concern when using an administrative database in a retrospective study is the unadjusted factors. The socioeconomic status (SES) has been widely studied as an important factor in the choice of surgery[7]. Patients with higher family income ($ 40,000) are more likely to receive breast reconstruction after mastectomy[8]. Surgeons are more likely to offer breast reconstruction to women with higher education[9], who may also be more likely to have greater access to medical surveillance[10]. In the SEER database, we only had information about the median family income and percentage of people with a bachelor’s degree in a county, which does not reflect the SES for each patient in the same county. Additionally, tissue reconstruction may lead to more surgical complications and is more likely to delay the delivery of adjuvant systemic therapy compared with implant reconstruction. Without detailed information about the adjuvant systemic therapy in the SEER database, we are still not sure whether the BCSS advantage of implant (vs. tissue) reconstruction was associated with the choices and delay of the chemotherapy treatments. Furthermore, body mass index(BMI), the HER-2 status, BRCA gene mutation, family history of breast cancer and type of implant reconstruction (immediate/staged procedure) were not available in the SEER database, which may be important limitations in our study. Women who get delayed reconstruction are more likely to get tissue reconstruction compared to those women who have immediate reconstruction due to changes in the skin with radiation.

### Implications of the study

The BCSS difference between reconstruction approaches (tissue/implant) might have some biological reasons[11]. Some studies have suggested an anticarcinogenic effect of silicone implants[12,13] and the potential role of adipocytes in promoting the growth of breast cancer[14,15]. Additionally, there are no guidelines or consensus for optimal screening program in patients after breast reconstruction. The implant is placed behind the pectoralis major muscle, which displaces the entire mastectomy site anteriorly; therefore, physical examination of the skin and chest wall is very effective for surveillance[16]. In contrast, tissue-reconstruction patients may need post-operative mammography screening because nearly a third of the recurrences cannot be detected by physical examinations alone in this setting[16]. Since our study revealed that there is only a BCSS difference in N2-3 patients but not in N0-1 patients, it would be important to reconsider whether different screening programs are needed for these two subgroups of patients.

### Summary

Implant (vs. tissue) reconstruction after mastectomy was associated with an improved BCSS in N2-3 breast cancer patients, which was not the case for N0-1 patients. A well-designed, prospective study is needed to further confirm these findings.

## Supporting Information

S1 Table Univariate analysis of risk factors for CSS. (XLS)

S2 Table Cox-regression models and the interaction between N-stage and reconstruction type. (XLS)

S3 Table Adjusted Cox-regression with propensity score included as a covariate. (XLSX)

S4 Table Baseline characteristics in patients with N2-3 diseases. (XLS)

S1 File Original clean data for analysis in STATA 13.0 version. (DTA)
